# Research on Formation of Microsatellite Communication with Genetic Algorithm

**DOI:** 10.1155/2013/509508

**Published:** 2013-09-02

**Authors:** Guoqiang Wu, Yuguang Bai, Zhaowei Sun

**Affiliations:** ^1^State Key Laboratory of Structural Analysis for Industrial Equipment, Dalian University of Technology, Dalian 116024, China; ^2^School of Aeronautics and Astronautics, Dalian University of Technology, Dalian 116024, China; ^3^Research Center of Satellite Technology, Harbin Institute of Technology, Harbin 150006, China

## Abstract

For the formation of three microsatellites which fly in the same orbit and perform three-dimensional solid mapping for terra, this paper proposes an optimizing design method of space circular formation order based on improved generic algorithm and provides an intersatellite direct spread spectrum communication system. The calculating equation of LEO formation flying satellite intersatellite links is guided by the special requirements of formation-flying microsatellite intersatellite links, and the transmitter power is also confirmed throughout the simulation. The method of space circular formation order optimizing design based on improved generic algorithm is given, and it can keep formation order steady for a long time under various absorb impetus. The intersatellite direct spread spectrum communication system is also provided. It can be found that, when the distance is 1 km and the data rate is 1 Mbps, the input wave matches preferably with the output wave. And LDPC code can improve the communication performance. The correct capability of (512, 256) LDPC code is better than (2, 1, 7) convolution code, distinctively. The design system can satisfy the communication requirements of microsatellites. So, the presented method provides a significant theory foundation for formation-flying and intersatellite communication.

## 1. Introduction

Compared to using large, heavy, single-mission satellites, resource integrations of several smaller and smarter satellites have significant benefits in engineering applications like distributed aperture remote sensing due to its advantage in performance, cost, and so forth [1]. To make use of recent advances in MEMS, robust microsatellites can be built [2–4]. In order to integrate the resources of autonomous and formation-flying groups of microsatellites effectively, the satellites must have good ability to communicate with each other. Autonomy represents minimal dependence on ground stations for communication purpose, so ISLs can be used to allow each satellite to share their individual information and use their integrated resources in order to achieve much more complex application goals. A novel way to perform space missions is to utilize the concept of using satellite clusters, which can cooperate with each other in order to achieve the function of single large-scale satellite. Every microsatellite can communicate with any other one to share processing, communications, payloads, and some other mission functions. Hence, the required function is to spread across all the satellites of the cluster, of which the aggregation can form a virtual satellite. 

 Many investigations of satellites flying in formation have been presented. A brief review includes ESA missions Proba 3, Darwin, SMART-3, TechSat 21, SSTL SNAP-1, and NMP ST5 [5–8]. The satellite formation consists of a group of satellites performing a uniform mission, which is usually difficult to be achieved by a single larger-scale satellite. Wang et al. [9] proposed an optimal virtual center selection for formation flying maintenance. Zeng et al. [10] proposed a method to evaluate the safety of formation flying satellites.

 Formation stability is a key technology for microsatellite formation communication system. For the problems of formation design, Becerra et al. [11] investigated the possibility that can obtain periodic or quasiperiodic natural relative motion; Schaub and Alfriend [12] used *J*_2_ theory to design the same order formation, but found that their design produced inconvenient constraint. Kasdin and Koleman [13] used the epicyclic orbital elements theory to investigate bounded, periodic orbits in the presence of various perturbations. Inalhan et al. [14] found the analytical expression of the initial conditions which can result in periodic motion based on the classical Tschauner-Hempel equations. Sabol et al. [15] discussed four kinds of formation designs based on Hill equation and analyzed their stability under various perturbations. The space circular formation and nadir circular formation designed with the passage of time could produce deviation, which cannot satisfy the mission requirements. In this paper, an improved optimizing design method based on genetic algorithm is proposed for space circular orbit formation in order to satisfy the performance requirements of intersatellite communication.

Firstly, the satellite links are designed, the equations of intersatellite link are presented, and a simulation is provided to confirm the correction of the equations. Secondly, the derivation process of an improved genetic algorithm is presented, and a simulation is provided to confirm the feasibility of the proposed algorithm. Finally, a formation microsatellite intersatellite communication system is proposed under the design formation orbit based on the improved genetic algorithm, and we discuss the results of simulation.

## 2. Links Equations

 The basic task for intersatellite communication is transferring information among satellites. So the design of the satellite links must satisfy the communication quality demands. The guide of links equation is given by

(1) $$\begin{array}{c} \frac{E_{b}}{N_{0}}=\frac{P_{t}L_{1}G_{t}L_{s}L_{a}G_{r}}{kT_{s}R}. \end{array}$$

If (1) takes dB as unit, the calculation of the links can be convenient. By this way, if the designers calculate the links parameter, it is only needed to do some addition and reduction operations:

(2) $$\begin{array}{c} \frac{E_{b}}{N_{0}}=P_{t}+L_{1}+G_{t}+L_{s}+L_{a}+G_{r}+228.6-10\;\text{lg}\;T_{s}-10\;\text{lg}\;R, \end{array}$$

where: *E*_*b*_/*N*_0_, *L*_1_, *G*_*t*_, *L*_*s*_, *L*_*a*_, and *G*_*r*_ take dB as unit; *P*_*t*_ takes dBW as unit; *T*_*s*_ takes K as unit; *R* takes bit/s as unit. BPSK modulation style is chosen here. And the space environment of microsatellite formation-flying is assured. At the beginning, the change of power along with different code rate is simulated. Then, an appropriate transmitted power can be chosen for microsatellite transmitter.

**Figure 1:**
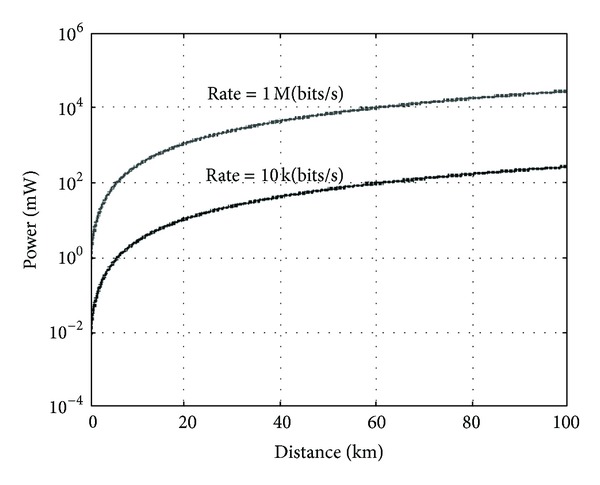
Simulated graph of transmitted power relative to distance.

The intersatellite RF links take *S* frequency. The transmitted frequency is 2 GHz, the error code rate request of links is 10*E* − 5. The full direction antenna is adopted. The communication distance can vary from 100 m to 100 km. The code rate must achieve 1 Mbit/s within 10 km and 10 kbit/s within the range of 10 km to 100 km, respectively. The feedback loss is 3 dB. The loss led by ionosphere is 0.7 dB. The remaining quantity of links is 5 dB. The simulation is given according to the links budget. The simulation results of the change of the transmitted power relative to distance are shown in Figure 1. It can be seen that, when the code rate is 10 kbps and the communication distance varies from 100 m to 100 km, the range of the power change is 250 mw, and that, when the code rate is 1 Mbps, the range of the power is 30 W. If the communication distance is within 10 km, the 250 mw transmitted power is enough. If 30% efficiency of the power output is adopted, the power of transmitter is less than 1 W. So the burden of satellites is not increased.

## 3. Design Method

 The change of intersatellite distance for microsatellites formation-flying can result in the change of space attenuation and satellite channel. Thus, the design of a steady formation order under various absorb impetus can reduce the complexity and the difficulty of intersatellite communication technology. The design of formation-flying order using Hill equation can appear as a biggish excursion under *J*_2_ absorb impetus. In order to solve this problem, an optimizing design method of space circular formation order based on an improved generic algorithm is proposed in this paper. Genetic algorithm is initially built from problems which could represent the solution set of the beginning process of a population. This process will result in a population-like natural evolution as kid generation population is more adapted to the environment than the previous generations. So after decoding of the previous generations, the optimal individuals can be treated as approximate to the optimal solution [6]. The method can keep formation order steady for a long time under various absorbs impetus. According to the task requirements, the restrict condition of the relative position and velocity can be gained under the Hill coordinate series when the formation order is designed. The optimizing objective function is the apiece average orbit root number of principal and subordinate satellites, with its relative excursion low enough under *J*_2_ absorb impetus. The relative position and velocity should be transformed to relative orbit root number during the optimizing process. The average orbit factors of satellite under *J*_2_ absorb impetus, which occur in long-term excursion, include perigee breadth angle, rise point of intersection equator longitude and aclinic close point angle. The size of excursion item is relative to semilong axes, eccentricity rate, and orbit obliquity:

(3) $$\begin{array}{c} \dot{\Omega }\left(t\right)=-\frac{3}{2}J_{2}n{\left(\frac{R_{e}}{P}\right)}^{2}\cos i,\\ \dot{\omega }\left(t\right)=-\frac{3}{4}J_{2}n{\left(\frac{R_{e}}{P}\right)}^{2}\left(5\;\cos^{2}i-1\right),\\ \dot{M}\left(t\right)=n+\frac{3}{4}J_{2}n{\left(\frac{R_{e}}{P}\right)}^{2}\sqrt{\left(1-e^{2}\right)}\left(3\;\cos^{2}i-1\right). \end{array}$$

Therefore, the optimizing objective function can be given as

(4) $$\begin{array}{c} J=\underset{x}{\min}\;K_{\Omega }{\left({\dot{\Omega }}_{d}-{\dot{\Omega }}_{c}\right)}^{2}+K_{\omega }{\left({\dot{\omega }}_{d}-{\dot{\omega }}_{c}\right)}^{2}+K_{M}{\left({\dot{M}}_{d}-{\dot{M}}_{c}\right)}^{2}, \end{array}$$

where

(5) ωd=ωc+δω,Ωd=Ωc+δΩ,Md=Mc+δM,Ω˙d=−32J2μ(a+δa)3×(Re(a+δa)(1−(e+δe)2))2cos⁡(i+δi),ω˙d=−34J2μ(a+δa)3×(Re(a+δa)(1−(e+δe)2))2(5 cos⁡2⁡(i+δi)−1),M˙d=−34J2μ(a+δa)3×(1+34J2(Re(a+δa)(1−(e+δe)2))2  ×(1−(e+δe)2)(3 cos⁡2⁡(i+δi)−1)).

If a formation includes many satellites, the optimizing objective function is given by

(6) $$\begin{array}{c} J=\underset{x}{\min}\overset{n}{\underset{i=1}{\sum }}K_{\Omega }^{i}{\left({\dot{\Omega }}_{d}^{i}-{\dot{\Omega }}_{c}\right)}^{2}+K_{\omega }^{i}{\left({\dot{\omega }}_{d}^{i}-{\dot{\omega }}_{c}\right)}^{2}+K_{M}^{i}{\left({\dot{M}}_{d}^{i}-{\dot{M}}_{c}\right)}^{2}. \end{array}$$

In this paper, the formation includes three satellites which consist of one host satellite and two subordinate satellites. The presented simulation is a generic algorithm based optimizing design method for space circular formation order. And the radius of space circular formation order is 1 km. During the optimizing process, the relative position, velocity, and orbit root numbers are:

(7) $$\begin{array}{c} \theta_{0}=-3.3001040e+002,\\ {\dot{y}}_{0}^{1}={\dot{y}}_{0}^{2}=-0.8328342. \end{array}$$

**Figure 2:**
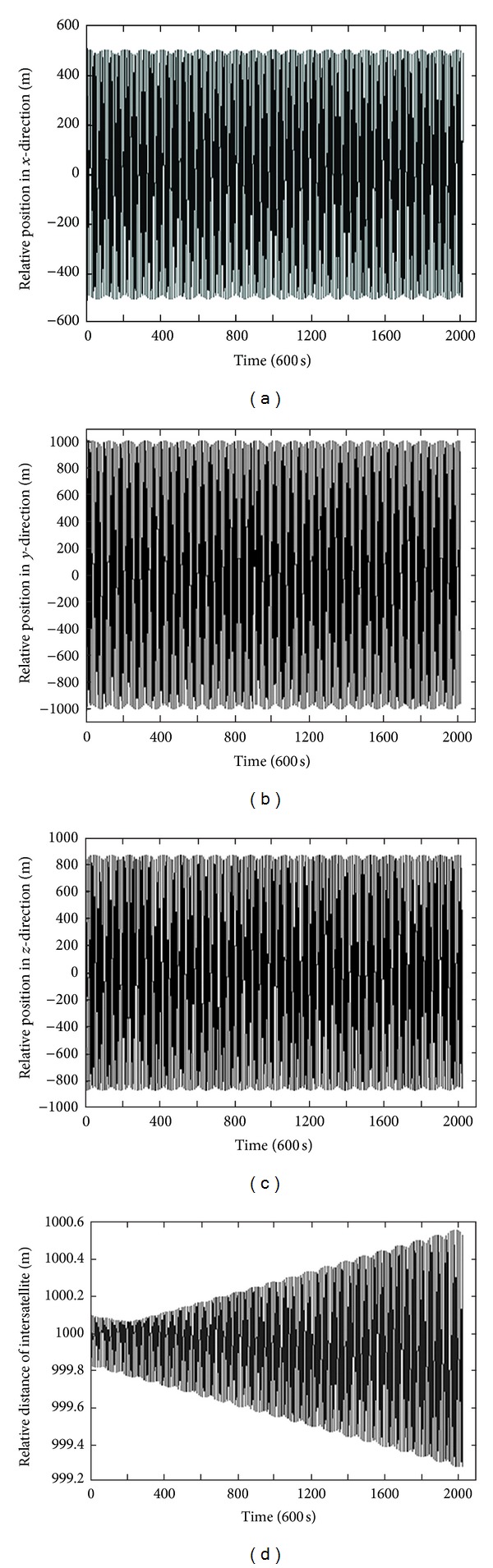
Simulation results of the first subordinate satellite.

**Figure 3:**
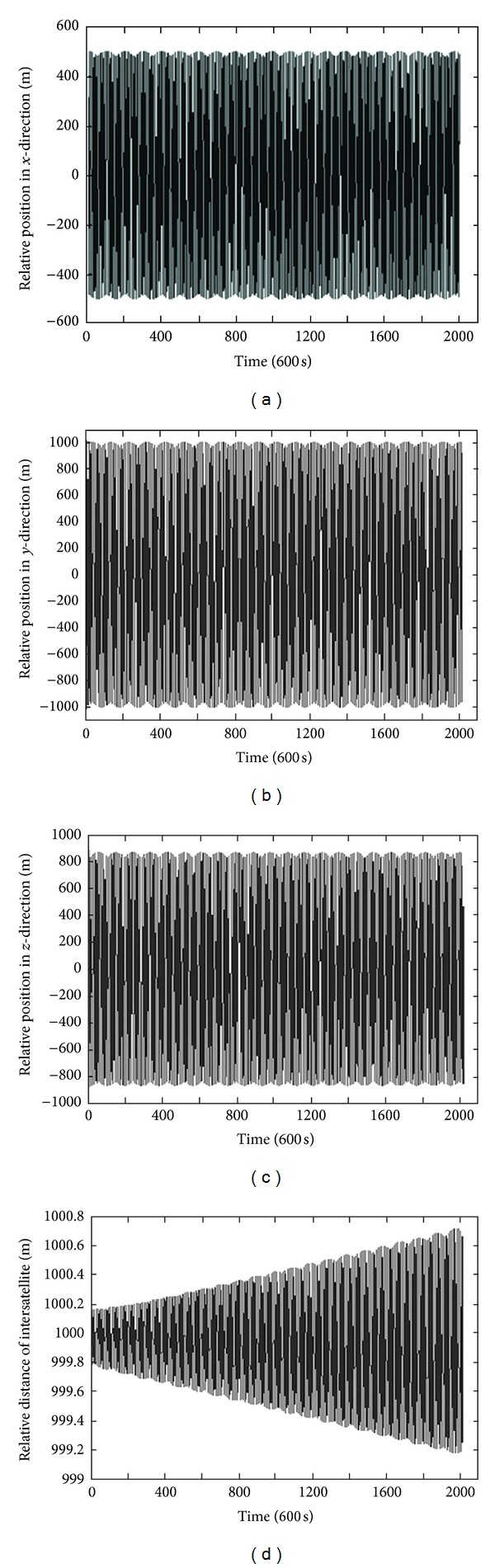
Simulation results of the second subordinate satellite.

Table 1 presents the orbit elements of formation flying satellites including several satellites. The results are shown in Figures 2 and 3 (i.e., in Figures 2 and 3: (a) the relative position in *x*-direction; (b) the relative position in *y*-direction; (c) the relative position in *z*-direction; and (d) the relative distance of intersatellite), where the former figure represents the first subordinate satellite, and the latter one represents the second subordinate satellite. The simulation time is 14 days, and the number of iterations is 120,960 (10 s per step). The simulation includes *J*_2_, *J*_3_, *J*_4_, and atmosphere resistance absorb impetus. It can be seen from Figures 2 and 3 that the relative position and relative distance excursion of the two satellites on the three axes are very small. These simulation results demonstrate that the proposed optimizing design method can design a long-time steady formation order under various absorb impetus.

Additionally, a comparison among the proposed generic algorithm and other optimizing methods has been taken in the presented work. Similar as what was found by Shi et al. [4], the presented method based on generic algorithm can obtain accuracy optimizing results with acceptable time requirement compared to the method based on another optimizing algorithm.

## 4. Simulation Results 

### 4.1. The Parameters of Direct Spread Spectrum Communication System

 Supposing that a formation including three microsatellites are flying on a same orbit and performing three dimension solid mapping for terra. Three microsatellites can get three solid images and process the error estimations of solid mapping. In such applications, the available load of each satellite is a camera. Mapping elevation data can be integrated with three satellites in a different perspective to get images of the same area. Compared with a single time-sharing satellite which maps the same area, a formation with three satellites has an advantage that the same area can be measured at the same time. So, the present microsatellites formation can measure not only the elevation of static objects, but also the elevation of dynamic objects. These features can provide a convenience to gain a long measurement baseline and a help to improve the accuracy of the survey and mapping. After the satellite gains the image data, its data transmission also has its own characteristics. 

Since every satellite has its own data-transmission system, the mapping data which solid mapping needs can transmit at the same time or in sequence according to the demands. It can decrease pressure on the data transmission and power consumption. Because the formation is fixed, free space loss can be calculated by it. Formation of the microsatellite system using satellite links between the S-band takes center frequency 2 GHz. Each satellite can link with any other satellites of formation. The distance between every two adjacent satellites is 1 km. The satellite antenna is an omnidirectional antenna. The intersatellite link parameters and budgets are shown in Tables 2 and 3. 

### 4.2. Formation of Microsatellite Intersatellite Communication System Model

**Figure 4:**
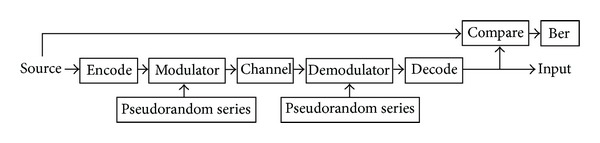
Diagram of intersatellite direct sequence spread spectrum communication system.

 The model of intersatellite communication system for formation microsatellites model is shown in Figure 4, where data source signal uses 1 MHz frequency random series, and spreading codes use 10 MHz frequency gold code.

**Figure 5:**
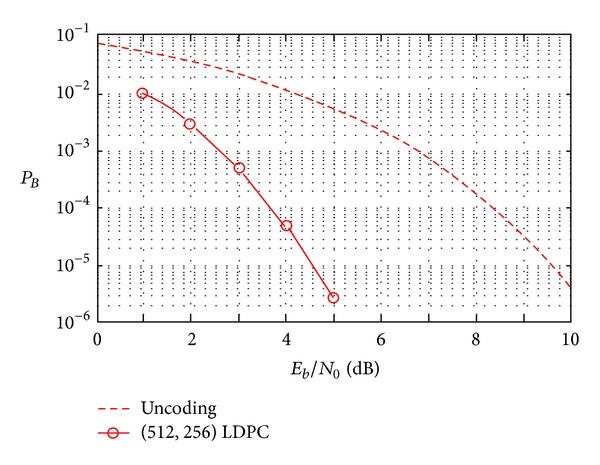
The bit error rate curve of the system (LDPC code) for the communication of a formation including three satellites.

The (512, 256) LDPC code is investigated under Gauss channel based on the platform. The code rate is 0.5, and the maximum number of iterations is 50. The bit error rates can be gotten under a different value of *E*_*b*_/*N*_0_ through changing the *E*_*b*_/*N*_0_ value of AWGN channel. The simulation results are shown in Figure 5.

**Figure 6:**
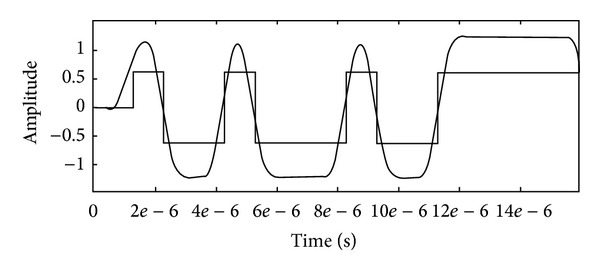
The export signals adding with the initial signals.

It can be seen from Figure 5 that, if the LDPC code is applied in the system, the bit error rates of the system can be improved obviously. This result can satisfy the requirements of intersatellite communication of a formation including three satellites. It can be seen from Figure 6 that the input wave matches well with the output wave. So, the result can also satisfy the requirements of the communication. Although the usage rate of available frequency is reduced under the transmission, the structure of transmitter can be predigested greatly, and the precision requirement of the fake code generator reduces greatly too. 

## 5. Conclusion

 It can be concluded from the simulation results of the intersatellite direct sequence spread spectrum communication system that LDPC code can improve the communication performance; the correct capability of (512, 256) LDPC code is better than (2, 1, 7) convolution code; and when the distance is 1 km and the data rate is 1 Mbps, the input wave matches well with the output wave. It can demonstrate that the proposed design system can satisfy the communication requirements of formations including many microsatellites. 

 The present work not only proposes an optimizing design method of formation order based on an improved generic algorithm, but also provides an intersatellite direct sequence spread spectrum communication system under the designed formation order with encouraging simulation results. So the presented work can provide a feasible theory foundation of the intersatellite communication for formation-flying microsatellites. For engineers, the satellite formation order and intersatellite communication system can be designed effectively and accurately with the convenient method proposed in this paper, which has significant potential benefits in aerospace engineering applications. 

## Figures and Tables

**Table 1:** Orbit elements of formation flying satellites including several satellites.

Orbital element	Host satellite	Difference of orbit element 1	Difference of orbit element 2
*a* (Km)	7555	−3.1816*E* − 7	3.2870*E* − 7
*e*	0.0001	−4.5999*E* − 5	6.0762*E* − 5
*i* (deg)	48	1.76130*E* − 6	−1.7613*E* − 6
ω (deg)	30	3.7782*E* + 1	−1.1876*E* + 1
Ω (deg)	30	−8.8383*E* − 3	8.8383*E* − 3
*M* (deg)	0	−3.7776*E* + 1	1.1870*E* + 1

**Table 2:** The parameters of intersatellite link.

Multiple methods	CDMA
Baseband filtering	Raised cosine
Modulation	BPSK
Data rate (Mb/s)	1
Center frequency (GHz)	2
Satellite launch power (mW)	250
Satellite *G*_*t*_ (dBW)	0
Satellite *G*_*r*_ (dBW)	0
Satellite receiving system noise temperature (K)	300
Coding and decoding manner	LDPC

**Table 3:** The budgets of intersatellite link.

Power (mW)	250
Satellite *G*_*t*_ (dBW)	0
Satellite *G*_*r*_ (dBW)	0
*L*_*a*_ (dBW)	0.7
*L*_1_ (dBW)	3
Noise temperature (K)	300
Link *E*_*b*_/*N*_0_ (dB)	9.6
Margin (dB)	5
Channel model	Gauss

## Nomenclature

*a*: Semilong axes
BPSK: Binary phase shift keying
*c*: Host satellite
CDMA: Code division multiple access
*d*: Slave satellite
*e*: Eccentricity rate
*E*_0_: Received energy per unit
ESA: European Space Agency
*G*_*t*_: Gain of transmitting antenna
ISLs: Intersatellite links
*J*_2_, *J*_3_, *J*_4_: Absorb impetus
*K*_*Ω*_, *K*_*ω*_, *K*_*M*_: Adding right coefficients
*L*_1_: Feed line loss
*L*_*a*_: Transmission path loss
*L*_*s*_: Propagation loss
LDPC: Low density parity check code [16]
LEO: Low earth orbit [17]
*M*: Aclinic close point angle
MEMS: Micro electromechanical systems
*n*: $\sqrt{u/n^{3}}$
*N*_0_: Noise spectrum density
*P*: $a\sqrt{1-e^{2}}$
*P*_*t*_: Transmitter power
*R*: Data rate
*R*_*e*_: A constant equal to 6378.136 km
RF: Radio frequency
*T*_*s*_: System noise temperature
$\Omega ,\dot{\Omega }$: Rise point of intersection equator longitude and its time derivatives
*ω*: Perigee breadth angle
*δM*, *δω*, *δΩ*: The relative orbit root number based on aclinic close point angle, orbit obliquitous, and rise point of intersection equator longitude, respectively.
